# Understanding the Pathophysiology of Cerebral Amyloid Angiopathy

**DOI:** 10.3390/ijms21103435

**Published:** 2020-05-13

**Authors:** Laura Gatti, Francesca Tinelli, Emma Scelzo, Francesco Arioli, Giuseppe Di Fede, Laura Obici, Leonardo Pantoni, Giorgio Giaccone, Paola Caroppo, Eugenio Agostino Parati, Anna Bersano

**Affiliations:** 1 Neurobiology Laboratory, Cerebrovascular Unit, Fondazione IRCCS Istituto Neurologico Carlo Besta, 20133 Milan, Italy; laura.gatti@istituto-besta.it (L.G.); francesca.tinelli@istituto-besta.it (F.T.); francesco.arioli@studenti.units.it (F.A.); 2 Cerebrovascular Unit, Fondazione IRCCS Istituto Neurologico Carlo Besta, 20133 Milan, Italy; emma.scelzo@istituto-besta.it (E.S.); eugenio.parati@istituto-besta.it (E.A.P.); 3 Unit of Neurology 5 and Neuropathology, Fondazione IRCCS Istituto Neurologico Carlo Besta, 20133 Milan, Italy; Giuseppe.DiFede@istituto-besta.it (G.D.F.); giorgio.giaccone@istituto-besta.it (G.G.); paola.caroppo@istituto-besta.it (P.C.); 4 Amyloidosis Research and Treatment Centre, Fondazione IRCCS Policlinico San Matteo, 27100 Pavia, Italy; l.Obici@smatteo.pv.it; 5 “Luigi Sacco” Department of Biomedical and Clinical Sciences, University of Milan, 20157 Milan, Italy; leonardo.pantoni@unimi.it

**Keywords:** cerebral amyloid angiopathy, amyloid beta protein, biomarkers, neuroimaging, outcome, small vessel disease, pathophysiology, treatment, dementia

## Abstract

Cerebral amyloid angiopathy (CAA), one of the main types of cerebral small vessel disease, is a major cause of spontaneous intracerebral haemorrhage and an important contributor to cognitive decline in elderly patients. Despite the number of experimental in vitro studies and animal models, the pathophysiology of CAA is still largely unknown. Although several pathogenic mechanisms including an unbalance between production and clearance of amyloid beta (Aβ) protein as well as ‘the prion hypothesis’ have been invoked as possible disease triggers, they do not explain completely the disease pathogenesis. This incomplete disease knowledge limits the implementation of treatments able to prevent or halt the clinical progression. The continuous increase of CAA patients makes imperative the development of suitable experimental in vitro or animal models to identify disease biomarkers and new pharmacological treatments that could be administered in the early disease stages to prevent irreversible changes and disease progression.

## 1. Introduction

Cerebral amyloid angiopathy (CAA), which is characterized by the accumulation of amyloid fibrils in the walls of small to medium-sized arterial blood vessels, and in capillaries of the central nervous system (CNS) parenchyma and leptomeninges, is a major cause of spontaneous intracerebral haemorrhage (ICH) in elderly people and an important contributor to age-related cognitive decline. The most common clinical presentations of CAA are ICHs, which occur predominantly in lobar sites and typically in the posterior regions. Other disease phenotypes include cognitive deficits and transient focal neurological events (TFNEs) or amyloid spells, which manifest with recurrent stereotyped brief episodes of focal negative neurological symptoms. Although the disease diagnostic confirmation is pathological, several neuroradiological features including lobar cerebral microbleeds, cortical superficial siderosis, cortical microinfarcts, white matter hyperintensities and enlarged peri-vascular spaces in semioval centres have been identified as possible disease features [1]. Of these lobar intracerebral haemorrhage, lobar microbleeds, and cortical superficial siderosis are considered so far the only disease biomarkers and are mandatory for CAA clinical diagnosis, according to the Boston modified criteria [2,3], although some serological and cerebrospinal fluid (CSF) biomarkers such as Aβ_42_ and Aβ_40_ levels have been also proposed for diagnostic purposes with controversial results [4,5].

The pathogenesis of CAA is largely unknown, although it is believed that in most cases the disease is due to an abnormal production or impaired clearance of the amyloid beta protein (Aβ), a cleavage product of the amyloid precursor protein (APP) leading to aggregation and accumulation in the walls of the small- and medium-calibre leptomeningeal and cortical arteries.

The incomplete knowledge of pathogenic drivers reflects in the lack of therapies able to slow or halt disease progression and severity. The aim of the present review is to discuss the key disease pathophysiological mechanisms and experimental models and dissect their relevance for understanding CAA and for the design of future research studies.

## 2. CAA Pathophysiological Mechanisms

### 2.1. Amyloid Generation and Clearance: The Role of the Neurovascular Unit

The most shared pathophysiological mechanism hypothesizes that sporadic CAA is caused by a mechanism of accumulation or impaired clearance of amyloid protein, mostly Aβ, an enzymatic product of the APP, in the walls of the leptomeningeal and small- and medium-calibre arteries [6,7]. CAA most severely affects occipital lobes, whereas the hippocampus, cerebellum and basal ganglia are less frequently involved. A neuropathological grading system (mild, moderate, severe) for CAA has also been proposed, depending on quantity of amyloid deposition and loss of smooth muscle cells (SMCs), focal vessel fragmentation and perivascular blood leakage [8]. Unlike Alzheimer’s disease (AD), in which the peptides usually deposited are Aβ_42_, the fragments that extend to the amino acid positions 39 or 40 (Aβ_39-40_) are mainly accumulated in CAA. The structural alterations of the vessels would be responsible for both ischemic lesions induced by the reduced blood supply and brain haemorrhages due to the fragility of the vessels [9]. Aβ would appear to gradually deposit in perivascular spaces inducing stagnation of interstitial fluids and leading to changes in cerebral microcirculation caused by chronic hypoperfusion. Moreover, alteration in vascular reactivity and in particular an impaired vasodilation have been identified early in the disease, before the onset of symptoms or the appearance of brain atrophy suggesting that they may be responsible of neurodegeneration [9]. Furthermore, these phenomena would seem to initiate and/or accelerate the neurodegenerative processes through multiple mechanisms, including the induction of oxidative stress, the further accumulation of Aβ and neuroinflammation [10].

**Figure 1:**
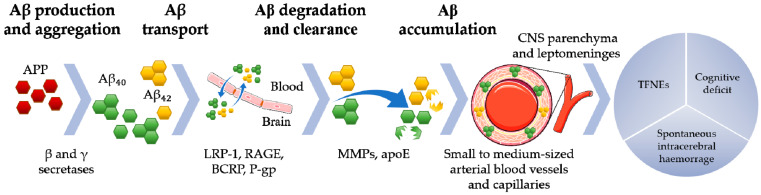
Amyloid precursor protein (APP) is cleaved in the amyloidogenic pathway by β and γ secretases to form amyloid beta (Aβ) Aβ_40_ and Aβ_42_ peptides. Aβ_40_ is the main peptide associated with cerebral amyloid angiopathy. These peptides are transported across the blood brain barrier (BBB) through membrane receptors and transporters (i.e., low density lipoprotein receptor-related protein 1, LRP-1; receptor advanced glycation end products, RAGE; breast cancer resistance protein, BCRP; permeability glycoprotein, P-gp) and degraded by specific enzymes, such as matrix metallopeptidases (MMPs) and apolipoprotein E (apoE). The accumulation of amyloid fibrils in the walls of the small- and medium-calibre leptomeningeal and cortical arteries may result from increased production and impaired transport and degradation of Aβ peptides. This aberrant pathway causes spontaneous intracerebral haemorrhage, cognitive decline and transient focal neurological events (TFNEs; CNS, central nervous system).

Recently, evidence has emerged in favour of mechanisms of impaired Aβ clearance linked to the integrity failure of the small vessels and blood-brain barrier (BBB) [6,7] (Figure 1). Initially described in 1997, the neurovascular unit (NVU) was presented as a functional domain in which cellular components such as neurons, astrocytic end feet, endothelial cells, vascular SMCs (VSMCs) and pericytes established a strict and intimate inter-communication. The concept of the NVU highlighted the interdependence between neurons and blood vessels in maintaining the brain homeostasis and in regulating the cerebral blood flow (CBF) according to the brain activity. In this sense, any damage to each of the NVU elements could potentially lead to cerebrovascular or neurodegenerative diseases [11]. Over the years, the role of its cellular components in the CAA has been extensively studied as abnormal alterations in the CBF following the overexpression of Aβ_40_ provided a first clue of the NVU malfunctioning in the disease [12]. The CBF has been demonstrated to be initially regulated by the dilation of pericytes in vivo, both under ischemic and healthy conditions, and a recent in vitro study pointing out the toxic effects of Aβ_40_ on this cellular population opened the way to questions on their implication in the CAA pathogenesis [13,14]. Indeed, pericytes have already been shown to respond to the astrocytic release of apoE by stopping the production of MMP-9, which would lead to BBB disruption if unregulated [15]. The astrocytic release of cytokines in the presence of Aβ and the consequent initiation of the inflammatory process was proposed to lead to the BBB destabilization through an increased production of matrix-degrading enzymes such as MMP-2 and MMP-9 [16,17]. The toxic effects of Aβ accumulation on the BBB integrity were further evidenced by the loss of tight junction proteins such as claudin-1, claudin-5, zonula occludens-1, occludin and recently confirmed in CAA by pointing out a reduced expression of the endothelial marker CD31 and an enhancement in the immunoreactivity for the leakage marker fibrinogen [18,19]. Astrocytes’ implication in the disease was also demonstrated by highlighting, both in mouse models and in human, an altered appearance together with changes in expression of several channels, which could at least partially explain the altered neuronal excitability witnessed in AD and CAA [20]. Moreover, the production of Aβ-degrading enzymes, such β-site amyloid precursor protein cleaving enzyme 1, neprilysin, insulin-degrading enzyme and angiotensin-degrading enzyme, has been shown to be a pivotal function of astrocytes in the disease [21]. In this sense, any change in the astrocytic reactivity could contribute to the CAA progression. Early accumulation of fibrillary Aβ_40_ in the tunica media and adventitia of the blood vessels is known to exert cytotoxic effects also on the surrounding VSMCs, leading to the cellular death as the disease progresses [22]. Being contractile cells, VSMCs were suggested to play a pivotal role in initiating the intramural periarterial drainage in order to facilitate the Aβ clearance and for this reason they were proposed as a novel target in the CAA prevention [23]. The development of CAA was recently proposed to be enhanced by the contextual loss of cholinergic innervation towards VSMCs, astrocytes and capillaries [24], but despite the necessity of considering the NVU as a whole entity, most experiments investigating its alterations in the CAA have been focused so far on single components rather than on how each part interacts with the others.

### 2.2. The Prion Hypothesis

Another intriguing hypothesis is represented by the progression of the disease through a template misfolding mechanism similar to prion diseases [25]. This suggestion is part of the more general view that misfolding proteins like α-synuclein, tau, Aβ and others share with the pathological form of the prion protein several ‘prion-like’ features that can be relevant for the pathogenesis of several neurodegenerative disorders. These features essentially include structural/conformational/biochemical variations, resistance to degradation by endogenous proteases, seeding ability, attitude to form neurotoxic assemblies, spreading and propagation of toxic aggregates, transmissibility of pathology to animal models [26]. Aβ seeding and spread of Aβ-related pathologies in animal models intracerebrally injected with synthetic Aβ or Aβ-containing human brain samples are increasingly reported and are considered to be based on prion-like mechanisms [27,28,29]. Recently, CAA was observed in the brain of a mouse model of AD even after a single intravenous injection of an AD patient’s brain extract [30], further supporting the hypothesis that CAA may—at least in part—occur following the prion protein -based pathogenic model [31]. Additional evidence in favour of this view comes from several reports on pathologically proven CAA in patients with iatrogenic Creutzfeldt-Jakob disease and in young individuals with early onset CAA, subjected to neurosurgical procedures performed some decades before the occurrence of CAA consisting of interventions with or without dura mater grafting and embolization of external carotid artery by dural extracts [32,33,34,35]. Very recently, we described the case of a young man who presented with clinical signs of a pathologically confirmed severe CAA, 28 years after a traumatic injury followed by neurosurgery during his early infancy [36]. Our report is in line with previous studies describing patients with early-onset iatrogenic CAA [37,38]. Some peculiarities of the neuropathological picture of these iatrogenic CAA cases, such as the biochemical composition of the Aβ deposits in the walls of the cerebral vessels which consist of both Aβ_42_ and A_β40_ species (without the strong predominance of Aβ_40_ peptide that usually characterize sporadic and genetic forms of CAA), suggest a link between iatrogenic CAA and the use of contaminated neurosurgical instruments or the exposure to dura mater containing Aβ seeds, similarly to what happen in iatrogenic Creutzfeldt-Jakob disease cases [39]. Despite the growing findings suggesting a prion-like modality of transmission at least in apparent iatrogenic forms of CAA, the prion-like hypothesis for CAA has yet to be substantiated by more in-depth studies. Indeed, although a prion nature of CAA is mainly suggested by evidence coming from transmission studies in animal models, the rigorous application of the prion concept to CAA likely requires to solve some issues concerning the existence of a real misfolding phase preceding aggregation of Aβ molecules, the explanation of the connection between seeding capacity and structural features of Aβ peptides involved in CAA, and the existence of strains of Aβ—fully matching the prion strain concept—conferring to its assemblies the ability to drive the pathobiology towards specific and well-defined clinic-pathological entities [26,40,41].

### 2.3. Genetic Factors Involved in Aβ-CAA Formation

The progress in the study of AD and the early identification of amyloid deposit in AD and Down syndrome led to the identification of an association between *APP* gene and Aβ peptide in CAA [42]. In the following years molecular analysis studies identified mutations in several factors (i.e., APP, preseninlin, cystatine C, British precursor protein) in rare families with hereditary cognitive impairment and cerebral haemorrhage. In most cases patients shared haemorrhagic and AD phenotypes.

#### 2.3.1. APP Genetic Variations Associated with Autosomal Dominant CAA

Mutations in the *APP* gene, mostly resulting in single amino acid substitutions, are responsible for autosomal dominant, early onset AD and/or CAA. Variants affecting residues within the Aβ region manifest with prominent or exclusive CAA, although the mechanisms behind the predominant vascular pathology are not clearly defined [43]. Three mutations, namely E693Q (Dutch), E693K (Italian) and L705V (Piedmont) are uniquely associated with severe amyloid angiopathy without neurofibrillary pathology or dense core Aβ plaques [44]. Other variants, like E693G (Arctic) and D694N (Iowa), manifest with both AD and CAA features. Additionally, increased copy number of the *APP* gene, as it occurs in early onset AD associated with gene duplication and Down’s syndrome, also result in severe Aβ-CAA [45]. 

Hereditary cerebral haemorrhage with amyloidosis-Dutch type (HCHWA-D), caused by the E693Q mutation, is the first and best characterized form of inherited CAA. In this form, severe Aβ deposition is observed in the cerebral and cerebellar meningeal arteries and cerebral cortical arterioles, which show loss of sSMCs, wall thickening and pathognomonic vessel-within-vessel (double barrel) features, reflecting severe vasculopathic changes. Vascular lesions occur more severely in the occipital lobes. Age at first stroke is around 50 years and severe cognitive deterioration commonly develops in those who survive haemorrhagic lesions [46]. Aβ_40_ is the major fibril component of vascular deposits, with wild-type and mutant peptides being equally represented. On the contrary, only the mutant Aβ_42_ isoform has been identified in amyloid fibrils in vessel walls [47]. Multiple molecular mechanisms have been advocated to play a role in the fibrillogenesis of Aβ mutants and in driving amyloid deposition primarily in vessels rather than brain parenchyma. In vitro, most Aβ variants show increased aggregation propensity compared to the wild-type counterpart and this is expected to be related to the change in net charge introduced by the mutated residue. Interestingly, a recent investigation of the aggregation kinetics of Aβ_42_ mutants has shown that mutations mostly affect the nucleation process and particularly increase the so called “secondary nucleation” step, which occurs at the surface of existing fibrils and has been associated with generation of toxic oligomeric species [48]. In animal models of HCHWA-D, increased Aβ_40_ versus Aβ_42_ neuronal production [49], impaired clearance [50] and higher affinity for VSMC membrane [51] and extracellular matrix components [52] have been related to its peculiar vascular tropism.

HCHWA-D represents a valuable model not only for dissecting the key pathological events underlying vascular Aβ deposition but also for identifying early biomarkers of disease onset and progression. By evaluating asymptomatic mutation carriers, it has been shown that reduced Aβ_40_ and Aβ_42_ levels in the CSF are present before the onset of symptoms, indicating early vascular amyloid deposition [53]. Moreover, vascular dysfunction as reflected by reduced cerebrovascular reactivity in the occipital lobe also anticipates clinical symptoms [54]. Whereas diffusion weighted magnetic resonance imaging (DW-MRI) seems not to be a sensitive imaging technique for early white matter changes [55], recently, the Pittsburgh compound B has been shown to image vascular amyloid in asymptomatic mutation carriers on positron emission tomography (PET) and to correlate with reduced CSF Aβ_40_ level [56], representing a potentially valuable marker for monitoring onset and disease progression also in the preclinical stage. 

#### 2.3.2. APOE Genotype in Sporadic Aβ-CAA

The *APOE* locus is a well-established determinant of sporadic CAA onset and severity. Definite evidence for a dose-dependent association with the ε4 allele was provided in a large meta-analysis including 3520 patients with pathologically proven CAA from 24 studies [57]. Further association of this allele with the risk of developing vasculopathic changes and severe CAA has also been reported [58]. Although both studies did not show a significant ε2 contribution, other observations point to the association of this allele with vessel cracking abnormalities that predispose to rupture and symptomatic haemorrhages. This was originally reported by Greenberg et al. [59] and later supported by the finding that ε2 is associated with larger ICH-related haematoma volume and poorer functional outcome [60]. Very recently, a strong association was confirmed between the ε2 allele and disseminated cortical superficial siderosis, that represents a specific MRI biomarker for more advanced CAA. This finding fosters the relationship between this genotype and the occurrence of vasculopathic changes driving small vessel bleeding [61]. It has therefore been speculated that the different contribution of the ε4 and ε2 alleles might be related to independent mechanisms:ε4 could enhance Aβ amyloid vascular deposition predominantly in the parenchymal vessels by promoting Aβ aggregation and by altering Aβ clearance and cellular metabolism, increasing the Aβ_40_:Aβ_42_ ratio, whereas the ε2 isoform could impact on bleeding risk and disease severity by affecting the degree and course of pathological changes occurring in amyloid-laden leptomeningeal and superficial vessels [61].

## 3. In Vitro Aβ-CAA Models

The incomplete disease knowledge and the absence of a treatment that could slow or halt the progression of the disease make necessary to establish CAA in vitro models enabling to (i) elucidate the complex molecular mechanisms behind the pathology and to (ii) develop potential pharmacological treatments. Due to abnormal production together with failure in clearance, the accumulation of Aβ is firmly involved in damages to BBB and small vessels [62,63]. Several works have studied the BBB properties to develop valuable tools to elucidate the mechanistic aspects of Aβ transport in the physio-pathological process, thus more likely predicting the in vivo context [64]. The excessive accumulation of Aβ could be caused by an aberrant expression and function of its receptors and transporters, such as receptor advanced glycation end products (RAGE), permeability glycoprotein (P-gp), low density lipoprotein receptor-related protein 1 (LRP-1), breast cancer resistance protein (BCRP) [65]. Candela P. et al. [66] planned a BBB in vitro model, co-culturing BCECs and rat glial cells on transwell system, to study the effects of Aβ_40_ and Aβ_42_ on the BBB permeability and their transcellular transport, focusing on the involvement of P-gp, BCRP and RAGE. The expression of the above mentioned Aβ transporters has also been studied in hCMEC/D3 cells, a model of human cerebral endothelial cells, treated with either fibrillar or oligomeric preparation of Aβ_40_ or Aβ_42_ [65]. The reduction of P-gp expression and transport activity, due to ubiquitin-proteasome pathway activation, was found also in freshly isolated rat brain capillaries when exposed to Aβ_40_, but not to aggregated Aβ_40_ or Aβ_42_ [67]. Moreover, Aβ accumulation has been reported to induce reactive oxygen species (ROS) production by NAHPH oxidase, resulting in altered tight junction expression and localization [68,69]. Aβ_42_ is toxic to hCMEC/D3 via RAGE-mediated ROS production and induces the downregulation of tight junction proteins such as occludin, claudin-5 and zonula occludens-1, as demonstrated by Carrano A. et al. [70]. In addition to hCMEC/D3, other brain endothelial capillary cell lines from various sources, such as rat (TR-BBB cells), bovine (BBCEC cells) and mouse (bEnd.3 cells) or engineered human hepatoblastoma (MEF cells) have been employed to build BBB in vitro models and to observe the Aβ cellular uptake, efflux and permeability in order to mimic the Aβ apical-to-basolateral permeability [71,72,73]. Indeed, the bidirectionally Aβ_40_ transport through the BBB is firmly controlled by membrane transporters and receptors, the main among them being LRP-1 and RAGE [74]. Aβ permeability from blood to CSF and vice versa was observed through transwell system consisting of bEnd.3 and HepG2 cell lines under hypoxia and incubated with Aβ_40_ to mimic the ischemic condition of CAA [75]. 

Several lines of evidence previously suggested that high-density lipoprotein (HDL) has potent anti-thrombotic, anti-oxidant, anti-inflammatory and cytoprotective functions [76], all of which may affect AD. Moreover, epidemiological evidence showed that AD risk can be attenuated by HDL levels [77,78,79]. Specifically, it has been demonstrated that HDL reduces AD risk by decreasing vascular Aβ deposition and inflammation [80,81]. Interestingly, in mouse models of amyloidogenesis, deficiency of apoA-I, which leads to low HDL levels, is reported to selectively increase CAA and cerebrovascular inflammation [82,83], whereas apoA-I overexpression reduces CAA and neuroinflammation [84]. Similarly, the systemic delivery of recombinant HDL or apoA-I Milano acutely decreases soluble brain Aβ levels, lowering CAA and neuroinflammation, respectively [85,86]. A novel human in vitro model of 3D perfusable bioengineered vessels was recently proposed to study CAA and Aβ-associated vascular inflammation [87,88]. It consists of engineered artificial arteries from human endothelial cells, SMCs and astrocytes, seeded on a tubular biodegradable polymer scaffold in a bioreactor chamber. To rebuild the CAA conditions, Aβ_40_ and Aβ_42_ have been added to the medium and flushed through the artificial arteries, thus studying Aβ accumulation, permeability and HDL-mediated clearance [87]. Since inflammatory stimulus activates endothelial cells increasing their interaction with leukocytes, this 3D biomimetic model has been exploited also to study the Aβ-induced peripheral blood mononuclear cell adhesion to human endothelial cells and to investigate the mechanisms by which HDL could protect cerebral vessels [88]. The same bioengineered model was very recently used to investigate how HDL particles enriched in apoE reduce Aβ accumulation in the vascular walls and attenuate endothelial Aβ-induced inflammation, thus providing new insights into the peripheral role of HDL in AD, in particular, the fraction of HDL that contains apoE [89]. Indeed, Robert J. and colleagues demonstrated that the anti-CAA and anti-inflammatory functions of HDL are mediated by distinct mechanisms, and they defined four distinct pathways that HDL uses to attenuate Aβ accumulation, namely: i) altering Aβ binding to collagen-I, ii) forming a complex with Aβ that maintains its solubility, iii) diminishing collagen-I protein levels produced by SMCs, and iv) attenuating Aβ uptake into SMCs that is associated with reduced low density LRP-1 levels [89]. Another important step will be the engineering of the perivascular space and the glymphatic flow to recapitulate better the pathological condition. A large branch of studies is focused on the amyloid fibril formation. It is a nucleation-dependent polymerization mechanism consisting of two steps: (i) the nucleation, in which Aβ monomers associate, and (ii) the extension, in which associated monomers form fibrils [90]. The biological membranes or other interfaces in the brain may influence Aβ amyloid fibril formation. In this context, Morinaga A. et al. [91] investigated the effect of air–water interfaces, agitation, plastic surface and convection on Aβ aggregation in vitro, monitoring the fluorescent emission of thioflavin T, an amyloid specific dye. An innovative system was developed to evaluate the effect of the basal membrane components and extracellular matrix proteins on the induction of Aβ nucleation, by mimicking perivascular drainage flow and the basal membrane surface in vitro. To study the fibril growth kinetics, Hasegawa K. et al. [92] used sepharose beads, as an inert stirrer in air-free well, conjugating them with Matrigel or other proteins, such as laminin, fibronectin, human serum albumin and IgG, following the thioflavin T fluorescence trend over time. With the same model was investigated also the effect of apoE and clusterin, proteins significantly increased in the vessels of symptomatic CAA patients, on the early phase of Aβ aggregation [93]. Exhaustive CAA in vitro models, which include all the cellular components, are needed to investigate the molecular pathways underlying Aβ accumulation and clearance, in order to identify diagnostic and prognostic markers and to provide new targets for therapeutic intervention [94].

## 4. Animal Models

Animal models are of great interest in studying mechanisms and potential treatments for CAA. To be “translational” and to impact on clinical practice, an animal model should reproduce at least one of the pathological processes seen in human CAA. A full translational model would permit prospective studies of the timescale and the sequence of the events during disease development. Moreover, it could serve to identify novel molecular, cellular and physiological mechanisms of the pathological process. Animal models would provide pre-clinical testing of safety profile, optimal dosing and time-scale of drugs and other interventions, for proof of concept studies. Finally, translational models could be useful for validation of clinical biomarkers and endpoints such as radiological or biological signatures [95]. Although CAA was first described almost a century ago, progress in deciphering its underlying pathological mechanisms has been hindered by the lack of reliable animal models [96]. Definite CAA can only be diagnosed by post-mortem neuropathological evaluation, whereas a diagnosis of CAA during life is established using clinical data and neuroimaging-based criteria that display high specificity but limited sensitivity. Moreover, human CAA research from early stage of the disease is difficult because when the condition is detected often the disease is already at a later stage [97]. For longitudinal studies, naturally occurring animal models, including cats, dogs and non-human primates can be used. Although vascular pathology in these models is similar to human CAA, ethical issues, long lifespan and low throughput make these models problematic for pre-clinical research. Since rodents generally do not develop CAA spontaneously, not even at very old ages, a variety of transgenic mouse models have been introduced that, similar to cerebral amyloidosis in humans, develop either Aβ-CAA only or both Aβ-CAA and parenchymal amyloid, or primarily parenchymal amyloid. In addition, alternative mouse models make use of a second stimulus, such as hypoperfusion or hyperhomocysteinemia to accelerate CAA.

### 4.1. Naturally Occurring Animal Models

Several naturally occurring animal models of CAA exist. However, as an example, cats do not represent a good model, because they display a late age of CAA onset and only exhibit limited vascular Aβ compared with parenchymal deposits [97]. Aged dogs represent a most suitable model because the Aβ is very similar in dogs and humans and dogs have a similar exposure to human diet and living environments [97,98]. Dogs may be a suitable model system in which to examine the consequence of CAA on cognition, although the late age of onset remains a major drawback [95]. Compared with human CAA, both Aβ_40_ and Aβ_42_ are found in non-human primates that are also a physiological relevant model due to their close homology to humans and spontaneous occurrence of CAA. However, the use of primates for large studies has been hampered by the late age of onset, high housing costs and—above all—by ethical considerations [97].

### 4.2. Transgenic Mouse Models

#### 4.2.1. APPDutch Mice

The APPDutch mice were primarily designed to study CAA and are the only murine model that develops significant Aβ-CAA with no parenchymal amyloid plaques [49]. These mice were generated by overexpression of the 751 isoform of human *APP* bearing the E693Q “Dutch” mutation under the control of the neuron-specific murine *Thy-1* promoter element. Similar to HCHWA-D, aged APPDutch mice develop vascular amyloid deposits at 22-25 months of age, mainly within the walls of leptomeningeal vessels followed by cortical vessels, indicating that the E693Q aa substitution is sufficient to target Aβ towards the vessel walls. Vascular deposition of Aβ40 is associated with thickening of the basement membrane and severe loss of SMCs, while the endothelial cell layer remains intact, similar to human CAA morphology [49]. A drawback of this model is the late age of onset, although the absence of parenchymal plaques makes APPDutch mice an attractive model of hereditary human CAA [96,97]. A very recent study employing APPDutch mice showed that Aβ reduction at early disease time points with β-site amyloid precursor protein cleaving enzyme 1 inhibitor largely prevents CAA, thus providing pre-clinical basis for Aβ-reducing treatments in patients at risk of CAA and in pre-symptomatic HCHWA-D [99].

#### 4.2.2. APP23 Mice

The APP23 transgenic mouse model was the first reported model to develop significant Aβ-CAA [100]. These mice overexpress human *APP751* with the Swedish double mutation under the control of the neuron-specific murine *Thy-1* promoter element, and they develop significant age-related CAA with an onset at 9-12 months, in association with neuroinflammation and haemorrhages [96,97]. The thalamus is an important site for CAA in APP23 mice, in contrast with human CAA [101]. However, these mice may serve as a suitable model for CAA type 1, because CAA frequently affects capillaries in APP23 mice.

#### 4.2.3. Tg2576 Mice

The Tg2576 (synonym APPSwe) mouse is the most popular and widely studied murine model in AD research [102]. These mice overexpress the human *APP695* gene with the Swedish double mutation under the control of the hamster prion protein promoter, thus they develop CAA and significant parenchymal amyloid plaques. CAA is a less prominent feature in Tg2576 mice compared with APP23 mice, thus such a model might be better to recapitulate AD associated with CAA than sporadic CAA [97]. Interestingly, the effect of the anticoagulant dabigatran has been recently tested in Tg2576 mouse model of aging and CAA, establishing pre-clinical evidence of absence of dabigatran-induced intracerebral haemorrhage and thus providing some reassurance for use of the anticoagulant in high risk populations [103].

#### 4.2.4. PDAPP Mice

These mice overexpress human *APP770* with the V717F Indian mutation under the control of *PDGF-β* promoter [104]. Aβ-CAA is less prominent in these mice as compared to age-matched Tg2576 mice, thus this model might be of interest to study AD-related CAA [97]. 

#### 4.2.5. Tg-SwDI Mice

This transgenic mouse model was primarily designed to study CAA, through the overexpression of human *APP770* containing the Swedish double mutation (K670N/M671L) and the Dutch and Iowa (D694N) mutations under the control of neuron-specific murine *Thy-1* promoter element [105]. CAA is first observed at 3 months of age and the accumulation of Aβ in vessels is accompanied by a loss of SMCs and apoptosis of vascular cells [106]. This model, by exhibiting CAA-associated cognitive impairment and a strong neuroinflammatory response, represents a unique and valuable model to study CAA type 1 [97].

#### 4.2.6. APP/London Mice

These mice overexpress the London mutant (V717I) of the human *APP695* gene, under the control of neuron-specific murine *Thy-1* promoter element [107]. APP/London (APP/Ld) mice develop CAA at an earlier age in comparison with APPDutch mice, although the absence of the typical CAA-associated intracerebral haemorrhages limits the usefulness of such a model [97].

#### 4.2.7. APP/PS1 Mice

The APP/PS1 mice overexpress human *APP* containing the Swedish mutation and *presenilin-1* with exon 9 deletion [108]. CAA development starts at 6 months of age and it is significantly more pronounced and severe—including microhaemorrhages and neuroinflammation—in female mice than in male ones [109]. A very recent study was aimed to in vivo assess, by two-photon microscopy, the formation of microhaemorrhages and their spatial relationship with vascular Aβ depositions in the surrounding microvascular network of aged APP/PS1 mice with mild-to- moderate CAA [110]. The findings suggest that the presence of vascular Aβ per se does not directly predispose vessels to leak, but that complex flow dynamics within CAA-affected vascular networks likely play a role. This in vivo approach allows the longitudinal assessments of the role of vascular dysfunction on the formation of spontaneous lesions at a single-vessel level, which is currently not feasible in humans [110].

### 4.3. Alternative Mouse Models

There are many tools to increase the usefulness of the above-mentioned animal models normally displaying moderate levels of vascular Aβ or developing CAA at a late age [97].

The induction of hypoperfusion in CAA models may accelerate and accentuate CAA pathology. Bilateral common carotid artery stenosis (BCAS) is a method frequently used to induce chronic hypoperfusion [111]. BCAS approach in Tg-SwDI mice produced a 26% reduction of cerebral blood flow and a significant increase in leptomeningeal Aβ accumulation [112], whereas in APP23 mice BCAS was able to increase CAA in small cortical and leptomeningeal vessels [113]. Similarly, induction of hypertension by angiotensin II resulted in increasing CAA both in APP-DSL mice and APPSwe/PS1L166P mice [114,115]. In addition, the induction of hyperhomocysteinemia, another risk factor for cerebrovascular diseases, was found to determine a shift of Aβ deposition from the parenchyma to the vasculature in APP/PS1 mice [116]. 

## 5. Treatment of CAA

Since no effective treatments are available as yet, disease management is mostly focused on the prevention of incident and recurrent ICHs, which is similar to the management of any spontaneous ICH [117]. In particular, a strict control of blood pressure and a careful evaluation and weighing of the risk and benefits of antithrombotics and anticoagulants are recommended, on the basis of the current literature [62,118,119,120,121]. However, since the results of randomized trials are not still available, patient management should be weighted individually based on potential risk and benefits. 

Very few clinical trials have been performed to specifically assess drugs limiting the accumulation of Aβ amyloid in CAA so far. Ponezumab (PF-04360365, Pfizer) is an anti-Aβ_40_ selective antibody developed to prevent or reverse Aβ aggregation and deposition. Although Ponezumab seemed to reduce Aβ deposition and to improve vessel function in transgenic mice [122], a phase 2 clinical trial showed that this drug neither improved vascular reactivity nor it influenced the number of microbleeds in humans [123]. Safety and tolerability of an anti-Aβ agent (NC-758, Cerebril, Bellus Health, Inc) was assessed (NCT00056238, phase 2 clinical trial) in patients with lobar haemorrhage related to possible or probable CAA. However, trials to assess the efficacy of this agent have not been published yet. 

## 6. Experimental Therapeutic Approaches

Several potential therapeutic targets are being explored. The inhibition of the phosphodiesterase 3A pathway is known to promote cerebral vessel wall protection and anti-thrombotic mechanisms thus representing a promising strategy to prevent both ischemic and haemorrhagic lesions in CAA. According to this, Cilostazol, a selective phosphodiesterase 3A inhibitor, has been shown to reduce Aβ_40_ deposits and to rescue cognitive decline in Tg-SwDI mice by promoting perivascular drainage of soluble Aβ_40_ [124] and by improving the lymphatic function [125]. However, the pharmacological inhibition of this pathway does not seem to alter cerebral microhaemorrhages/cerebral microbleeds development in CAA mouse models [126]. Inhibiting Aβ assembly would be another potential approach to treat CAA. Recent advances showed that taxifolin, a bioactive catechol-type flavonoid with antioxidant properties, inhibits Aβ aggregation in vitro and improves CBF, facilitating amyloid clearance in the brain and suppressing cognitive decline in a mouse model of CAA [127]. Taxifolin also suppress Aβ production and modulate proinflammatory microglial phenotypes [128]. Since one proposed pathogenic mechanism of CAA is that inefficient Aβ clearance leads to abnormal Aβ accumulation in the brain and vessels, several therapeutic interventions have been tested in CAA animal models by enhancing Aβ clearance and drainage systems. Aβ-degrading enzymes such as neprilysin, angiotensin-converting enzyme, insulin degrading enzymes, and cathepsin play an important role in Aβ clearance and reduce the damage of Aβ to VSMCs. For instance, the upregulation of neprilysin (i.e., through gene therapy or dual-specificity tyrosine phosphorylation-regulated kinase 1A inhibition) has been proved to reduce Aβ concentration in mice [129]. It is also well-known that Aβ accumulation in the cerebral vessels interferes with the BBB ability to clear Aβ from the CNS [130]. Aβ transportation through the BBB is mediated by multiple receptors (i.e., RAGE) and usually results in endothelial cell oxidative stress and expression of proinflammatory cytokines which finally leads to cell apoptosis, inflammatory response and vascular dysfunction. Therefore, BBB Aβ receptors have been considered as potential therapeutic targets for AD. Indeed, the inhibition of RAGE-ligand interaction (i.e., by using soluble RAGE or anti-RAGE antibodies) suppress the accumulation of Aβ in brain parenchyma and reduces cerebrovascular vasoconstriction and neurovascular stress in transgenic mouse models [131,132]. L-norvaline, an isoform of the common amino acid valine with anti-inflammatory properties and the ability to inhibit arginase, has been shown to decrease the rates of BBB permeability, amyloid angiopathy, microgliosis, and astrodegeneration [133]. The meningeal lymphatics constitute an alternative route for Aβ clearance. In AD mice models, the induction of dural lymphangiogenesis has been shown to facilitate Aβ clearance, suggesting the potential of this strategy also for CAA. Besides, *APOE* alleles influence CAA pathogenesis by interfering with Aβ aggregation and clearance. As a result, molecules that interfere with the Aβ/apoE interaction are expected to modulate the amount of parenchymal and vascular Aβ accumulation. This hypothesis was confirmed in TgSwDI mice with extensive CAA by using the peptide Aβ12-28P, which reduced vascular amyloid deposits [134,135], while another peptide compound, CPO_Aβ17-21P, has been shown to have a higher efficacy and safety in APP/PS1 Tg mice [136]. Immunotherapy using anti-APOE antibodies, and small molecule inhibitors or gene editing strategies developed to drive production of APOE2 expression or to convert APOE4 to APOE2 or APOE3 are also potential therapeutic strategies [137,138,139,140]. As the accumulation of Aβ in the arterioles and capillaries might activate the complement system thus producing a chronic inflammatory response, another interesting target is represented by the complement-related clearance system [141]. Recent studies also underlie the ability of the endothelial nitric oxide to inhibit amyloidogenic processing of APP in both human cells and mouse cerebrovascular tissue [142] and of the angiotensin receptor blockers (i.e., intranasal losartan) to induce Aβ reduction, neuroprotection and neurogenesis. Since the absence of tau is thought to prevent synaptic dysfunction induced by amyloid oligomers in a mouse model of Familial Danish Dementia [143], tau level modulation has also been suggested as a therapeutic approach to prevent neurodegeneration associated with CAA. Finally, a recent study reported that the administration at CAA onset of an inhibitor of β-site APP-cleaving enzyme 1 not only decreases Aβ production but also significantly delays the development and progression of CAA and associated pathologies [99]. These findings stress the need to develop new pharmacological treatments that could be administered in the early stages of the disease to prevent irreversible related changes and stop disease progression. However, most evidence on potential treatments for Aβ-related diseases comes from AD patients and models, and further studies are needed to assess their role in the treatment of CAA.

## 7. Conclusions

The understanding of CAA pathogenesis is still largely incomplete. Since Aβ peptides can be degraded by proteolytic enzyme or be cleared via perivascular spaces by BBB or lymphatic system, the impairment of one or more of these mechanisms can induce the disease by Aβ deposition in the basement membranes of small vessel disease. However, although one of the putative mechanisms of CAA development is an imbalance between production and clearance of Aβ, it cannot explain the whole disease pathological spectrum as well as how the common soluble form of Aβ adopt an altered conformation and what are the mechanisms conditioning the disease progression. Processes involving a template misfolding mechanism similar to prion diseases as well as the impairment of NVU including change in the astrocytic reactivity or the proinflammatory and cytotoxic effect of the accumulated fibrillary Aβ_40_ in the tunica media and adventitia of the blood vessels, have been proposed to explain the disease dramatic progression. However, which mechanisms or pathways are really responsible for CAA development remains to be elucidated. The poor understanding of the disease pathophysiological mechanisms reflects the lack of therapies able to limit the Aβ deposition and the disease progression. Despite the first promising results from Ponezumab, an anti-Aβ40 selective antibody developed to prevent or reverse Aβ aggregation and deposition in transgenic mice [122], the efficacy of this treatment has not been demonstrated in humans [123] and other efficacy trials have not been published yet. Therefore, at present time the management of CAA is not different from the management of any common ICH. Given the increasing number of CAA patients, better understanding the natural history and pathophysiology of the disease and developing a treatment for CAA remain a priority. While a huge amount of clinical research is focusing in identifying neuroradiological disease diagnostic and progression markers, experimental animal models seem critical to improve our understanding of CAA. The spontaneous development of CAA in a variety of animals resembling sporadic CAA in elderly humans makes these models attractive. However, the relatively late age of CAA onset, high costs and ethical considerations limited their use in CAA research. Transgenic mouse models displaying a variable degree of CAA, short life span, low expense and easy genetic manipulations have emerged more recently, thus providing us with some key findings in the disease pathophysiology. However, since no single animal model for CAA completely resembles human CAA, the integration between experimental results and clinical data from large observational studies are mandatory to understand the mysteries of CAA and to develop effective therapeutic strategies [144].

## Abbreviations

CAA	Cerebral amyloid angiopathy
CNS	Central nervous system
ICH	Intracerebral haemorrhage
Aβ	Amyloid beta protein
APP	Amyloid precursor protein
TFNEs	Transient focal neurological events
AD	Alzheimer’s disease
DW-MRI	Diffusion weighted magnetic resonance imaging
PET	Positron emission tomography
CSF	Cerebrospinal fluid
SMCs	Smooth muscle cells
BBB	Blood-brain barrier
LRP-1	Low density lipoprotein receptor-related protein 1
RAGE	Receptor advanced glycation end products
BCRP	Breast cancer resistance protein
P-gp	Permeability glycoprotein
MMPs	Matrix metallopeptidases
apoE	Apolipoprotein E
HCHWA-D	Hereditary cerebral haemorrhage with amyloidosis-Dutch type
NVU	Neurovascular unit
VSMCs	Vascular smooth muscle cells
CBF	Cerebral blood flow
ROS	Reactive oxygen species
HDL	High-density lipoprotein
BCAS	Bilateral common carotid artery stenosis
